# Facile one-pot synthesis of CuCN by pulsed laser ablation in nitrile solvents and mechanistic studies using quantum chemical calculations

**DOI:** 10.1038/s41598-021-93768-7

**Published:** 2021-07-13

**Authors:** Talshyn Begildayeva, Ahreum Ahn, Shreyanka Shankar Naik, Seung Jun Lee, Jayaraman Theerthagiri, Tae Ho Kim, Myong Yong Choi

**Affiliations:** 1 Core-Facility Center for Photochemistry & Nanomaterials, Department of Chemistry (BK21 FOUR), Research Institute of Natural Sciences, Gyeongsang National University, Jinju, 52828 Republic of Korea; 2 Center for Supercomputing Applications, Korea Institute of Science and Technology Information, 245 Daehak-ro, Daejeon, 34141 Republic of Korea

**Keywords:** Chemical engineering, Photochemistry, Physical chemistry, Theoretical chemistry

## Abstract

Binding energies of different nitrile solvents and their utilization for CuCN formation were investigated through quantum chemical calculations. A pulsed laser ablation in liquid (PLAL) method for CuCN synthesis was developed herein. Initially, the interaction between the pulsed laser and the Cu-target generated Cu-ions and electrons at the point of contact. The laser beam also exhibited sufficient energy to dissociate the bonds of the respective solvents. In the case of acetonitrile, the oxidized Cu-ions bonded with CN^−^ to produce CuCN with a cube-like surface structure. Other nitrile solvents generated spherically-shaped Cu@graphitic carbon (Cu@GC) nanoparticles. Thus, the production of CuCN was favorable only in acetonitrile due to the availability of the cyano group immediately after the fragmentation of acetonitrile (CH_3_^+^ and CN^−^) under PLAL. Conversely, propionitrile and butyronitrile released large amounts of hydrocarbons, which deposited on Cu NPs surface to form GC layers. Following the encapsulation of Cu NPs with carbon shells, further interaction with the cyano group was not possible. Subsequently, theoretical study on the binding energies of nitrile solvents was confirmed by highly correlated basic sets of B3LYP and MP2 which results were consistent with the experimental outcomes. The findings obtained herein could be utilized for the development of novel metal–polymer materials.

## Introduction

The production of metal cyanides has attracted considerable interest due to their commercial applications in different industries. For instance, CuCN and AgCN are employed in the fields of pharmacology, gas production, mining, photography, and metal plating^1^. CuCN has gained particularly wide recognition owing to its unique characteristics, including luminescence, bioactivity, long-range magnetic order, and electron transfer properties^2^. Moreover, CuCN is also valuable in the crystal engineering field. The CN group acts as a conservative connector to produce a linear –Cu–CN– chain in CuCN. Cu^+^ ions favor tetrahedral or trigonal coordination in the presence of pyridyl and cyanide groups. This offers the possibility of expanding the arrangement of framework structures due to the strong coordinating properties of the ligands as well as their ability to act as connectors between various metal centers^3^. The structural properties of CuCN and AgCN are relatively similar. Both compounds exist in the form of more than one polymorph. Unlike other cyanides, they are presumed to form –Cu–CN–Cu–CN– chains^4,5^. The basic structure of CuCN exhibits static and large thermal displacement of the chain composed of CN or NC groups, which makes the structure formation problematic^6^. Despite the existence of numerous conventional synthetic routes for the preparation of metal cyanides, they mostly utilize toxic anions (e.g., cyanide), which can result in serious health issues affecting the nervous, vascular, cardiac, visual, metabolic, and endocrine systems^7^.

Recently, an environmentally friendly approach called pulsed laser ablation in liquid (PLAL) has been proposed as an attractive alternative to the typical chemical methods. The advantages of this surfactant-free technique include the lack of toxicity, speed, and absence of byproducts; therefore, it can be applied for the production of various nano- to submicron-sized materials^8^. Moreover, PLAL is highly energy efficient and can be employed for the preparation of inorganic/organic colloids without the necessity for chemical reducing agents. PLAL enables the regulation of various properties of materials, e.g., their shape, size, crystalline phase, and chemical composition, by controlling the working parameters, such as the laser wavelength and power, time as well as reaction solvent^9–12^. Focusing the intense pulsed laser on the surface of the desired target immersed in a liquid medium results in the creation of cavitation bubbles, which rapidly expand and collapse within a few microseconds to enable the nucleation and formation of the expected materials^13^. In this work, we employed PLAL in acetonitrile as a facile one-pot technique for the synthesis of CuCN. Considering potential applications, particles, which are stable in organic solvents, are desired because they can be used for the fabrication of novel valuable coordination polymers and other hydrophobic materials.

The present study investigated the conditions of PLAL of Cu in various organic nitrile solvents. The process resulted in the selective and efficient formation of CuCN and graphitic carbon (GC)-encapsulated Cu nanoparticles (NPs). During PLAL in various nitrile solvents, such as acetonitrile, propionitrile, and butyronitrile, the optimum laser wavelength was adjusted to 355, 532, and 1064 nm, respectively. The conducted theoretical studies provided insights into the solvent structures, binding energy, and bond dissociation energy during the reaction. The mechanistic studies based on the obtained results revealed that the laser prompted the fragmentation of acetonitrile into CH_3_^+^ and CN^−^. The latter bound to Cu ions, generating CuCN. Intriguingly, the production of cubic-structured CuCN was favored only in acetonitrile, while other highly ordered propionitrile and butyronitrile solvents generated spherically-shaped Cu@GC NPs. The synthesized CuCN and Cu@GC were characterized by various analytical techniques, including X-ray diffraction (XRD), Fourier transform (FT) Raman spectroscopy, field emission scanning electron microscopy (FE-SEM), energy-dispersive X-ray spectroscopy (EDS), and transmission electron microscopy (TEM). The evaluation confirmed the rapid synthesis of CuCN with a unique surface structure. The outcomes reported herein could be used for the production of new coordination/polymer materials for applications in the fields of catalysis, sensing, and electronics.

## Experimental section

### Materials

The Cu plate (≥ 99.99%) was purchased from Sigma-Aldrich, USA. Acetonitrile (high-performance liquid chromatography [HPLC] grade, 99.8%) was obtained from Daejung Chemicals & Metals Co., Ltd, South Korea. Propionitrile (> 99.0%) and butyronitrile (> 99.0%) were acquired from TCI, Japan. All materials were used as received.

### Synthesis of CuCN by PLAL

**Figure 1:**
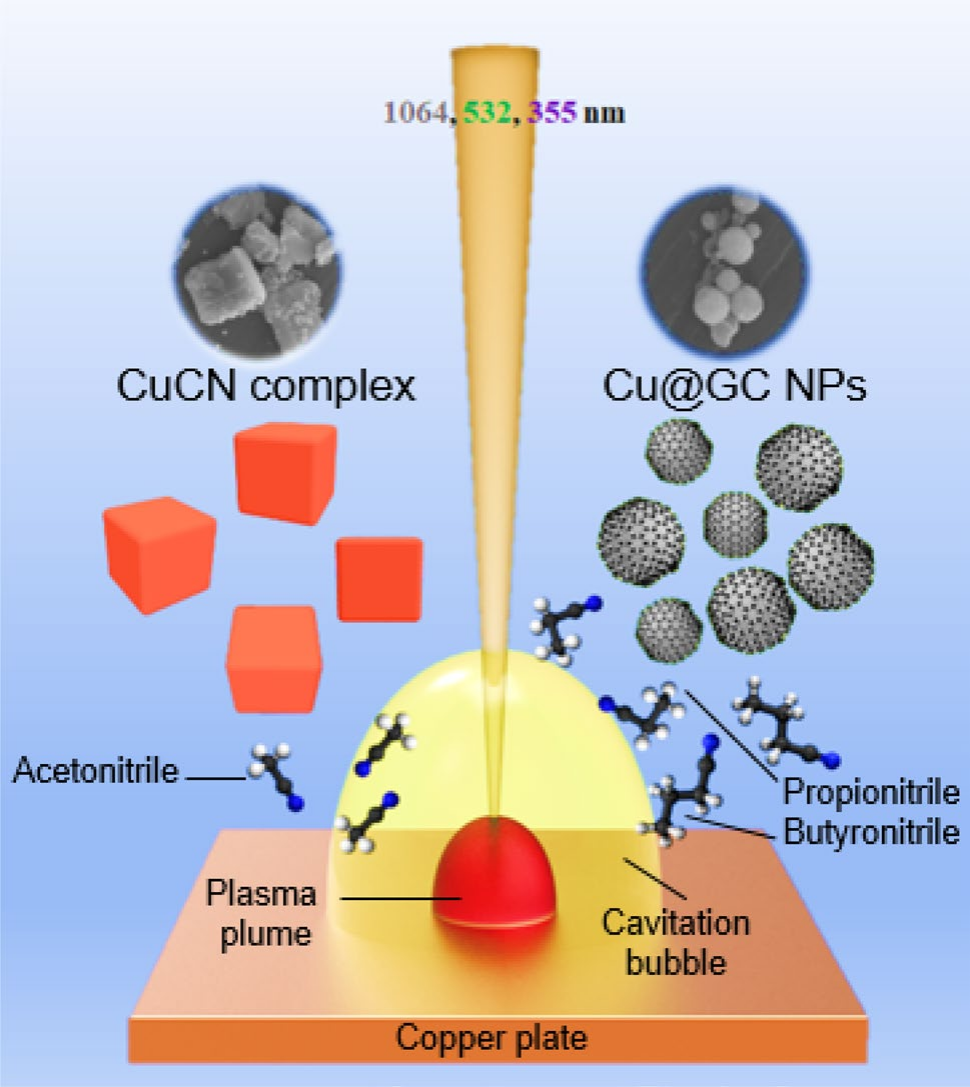
PLAL (1064, 532, and 355 nm) of a Cu target in various nitrile solvents (acetonitrile, propionitrile, or butyronitrile) leading to the formation of cubic CuCN and spherical Cu@GC.

The detailed experimental setup is illustrated as a schematic diagram in Fig. 1. A Cu plate was immersed in a vial containing 10 mL of a nitrile solvent (acetonitrile, propionitrile, or butyronitrile). Subsequently, the PLAL process was performed using a Q-switched Nd: YAG nanosecond laser exhibiting different wavelengths (355, 532, and 1064 nm) at a fixed pulse energy of 80 mJ (Continuum Surelite, SLII-10) for 10 min. To focus the laser beam on the surface of the Cu plate, a focusing lens of *f* = 30 mm was used. The final CuCN product was collected by centrifugation of the obtained colloidal solutions and dried at ambient temperature.

### Material characterization

To evaluate the crystallinity of the prepared materials, XRD analysis was conducted (Bruker, D8 Advance A25) using Cu Kα radiation (λ = 1.54 Å) in the 2θ° range of 20°–60°. The surface morphology, shape, and chemical composition of the synthesized materials were studied by SEM (TESCAN, CLARA) and EDS. Raman spectra were recorded employing a micro-Raman spectrometer with a 632.8 nm He–Ne laser beam as the excitation source (LabRAM HR800, HORIBA, Jobin–Yvon). TEM images were obtained on a 300 kV TF30ST (FEI) microscope. Quantum chemical calculations were performed using the Gaussian 16 program^14^. Geometry optimization and relative energy calculations for solvents (acetonitrile, propionitrile, and butyronitrile) were conducted by employing a 6-311++ G(d,p)^15^ basis set using the following two methods: B3LYP^16^ and MP2 (second-order Moller–Plesset perturbation theory)^17^. The binding energies of the solvents (acetonitrile, propionitrile, and butyronitrile) were corrected for the basis set superposition error (BSSE)^18–20^ using the counterpoise method. The obtained results were further corrected with zero-point vibrational energy.

## Results and discussion

### XRD analysis

**Figure 2:**
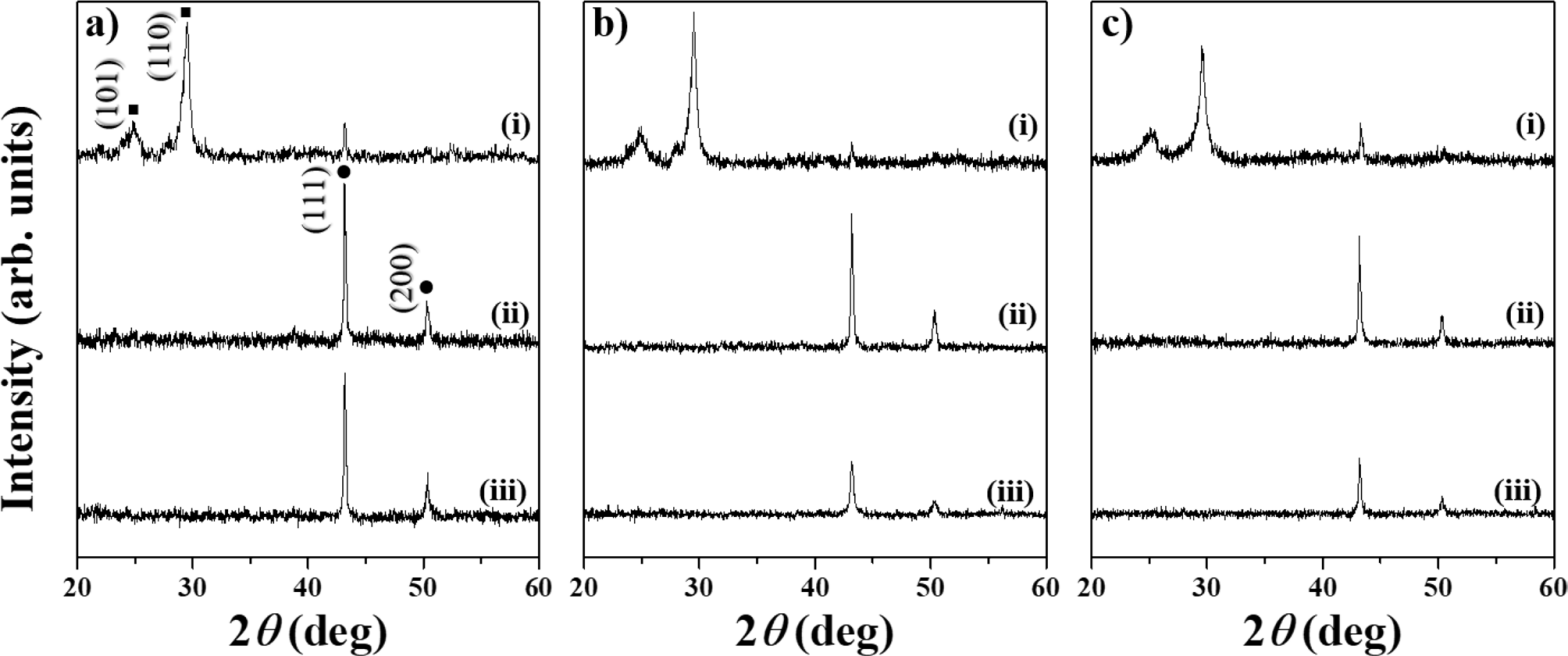
XRD patterns of samples synthesized by PLAL of a Cu plate in (i) acetonitrile, (ii) propionitrile, and (iii) butyronitrile at different laser wavelengths of (**a**) 355, (**b**) 532, and (**c**) 1064 nm. Characteristic peaks of metallic Cu at 43.2° and 50.4° denoted as (•) were consistent with JCPDS #01-085-1326. The CuCN peaks at 24.9° and 29.5° (JCPDS #96-110-1019) are indicated by (▪).

The formation of materials and their crystallinity were studied by XRD. The XRD patterns of the samples prepared by PLAL of a Cu plate at 355, 532, and 1064 nm in different nitrile solvents are illustrated in Fig. 2a–c. Ablation of the Cu plate at 355, 532, and 1064 nm in propionitrile and butyronitrile led to the formation of Cu NPs. The peaks at 43.2° and 50.4° were consistent with face-centered cubic copper NPs (JCPDS #01-085-1326)^21^. These peaks coincided with the diffraction of the (111) and (200) planes, respectively. The patterns of the Cu plate ablated at all wavelengths in acetonitrile revealed additional strong peaks at 25.1° and 29.5°, which were ascribed to the rhombohedral crystal phase of CuCN (JCPDS #96-110-1019). These peaks were indexed to the (101) and (110) crystallographic planes^22^. In addition to CuCN, a trace amount of pure metallic Cu NPs was present in the sample synthesized in acetonitrile, which was confirmed by weak diffraction peaks. However, the amount of Cu NPs in this sample was negligible compared to those in the propionitrile and butyronitrile samples.

### FT-Raman analysis

**Figure 3:**
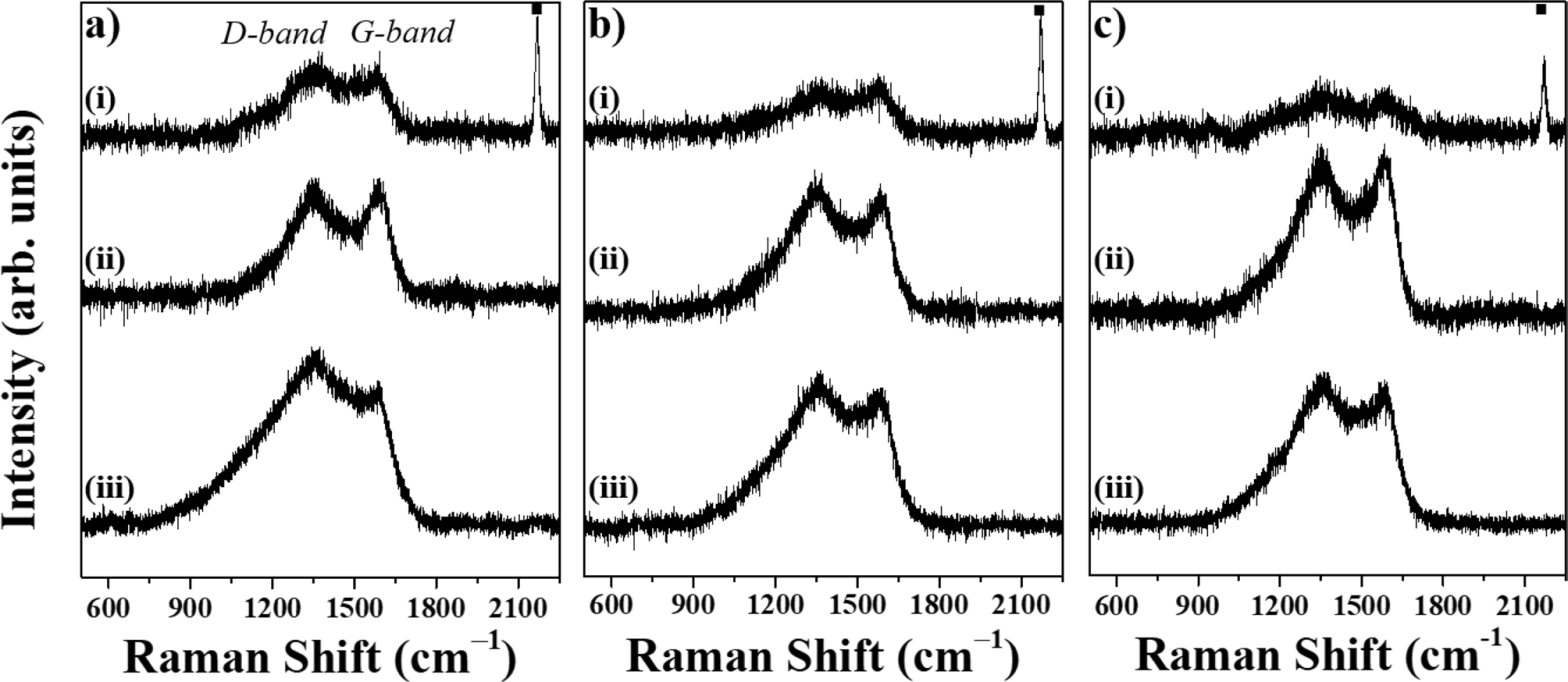
FT-Raman spectra of samples synthesized by PLAL of a Cu plate in (i) acetonitrile, (ii) propionitrile, and (iii) butyronitrile at different laser wavelengths of (**a**) 355, (**b**) 532, and (**c**) 1064 nm. Peaks at 1350 and 1590 cm^−1^ correspond to the D- and G-bands of the sp^2^ hybridized carbon, respectively. They were attributed to GC shells on the surface of spherical Cu NPs. The characteristic CuCN peak at 2170 cm^−1^ is indicated by (▪).

To further identify the molecules and intermolecular bonds, the samples prepared by PLAL of a Cu plate in various nitrile solvents were analyzed by FT-Raman spectroscopy. The FT-Raman spectra of all samples obtained at different laser wavelength (355, 532, and 1064 nm) are shown in Fig. 3a–c. As it can be seen, all spectra exhibited D- and G-bands, which are characteristic of GC. Thus, the D-band originated from a second-order process and described disordered carbon. The presence of GC layers was confirmed by the G-band arising as a result of the Raman scattering process in graphene^23^. The existence of D- and G-bands indicated the formation of pure metallic Cu NPs covered with GC layers during PLAL in nitrile solvents. More pronounced peaks were detected for the samples synthesized in propionitrile and butyronitrile, suggesting the generation of a high amount of Cu NPs. Notably, the spectra of the samples ablated in acetonitrile at 355, 532, and 1064 nm displayed an additional distinctive peak at 2175 cm^−1^^24^. This peak was attributed to the copper(I) cyanide species. The ν(CN) mode consists of A_1g_ + E_g_ + T_1u_, where A_1g_ is a high intense Raman active mode and T_1u_ is an IR active mode^25^. The C $\equiv$ N^−^ stretching vibration mode is commonly used as a structural and environmental probe in a variety of applications and can be found in the high frequency region of around 2000 cm^−1^^25,26^. The observed FT-Raman results were in good agreement with the XRD analysis.

### SEM analysis

**Figure 4:**
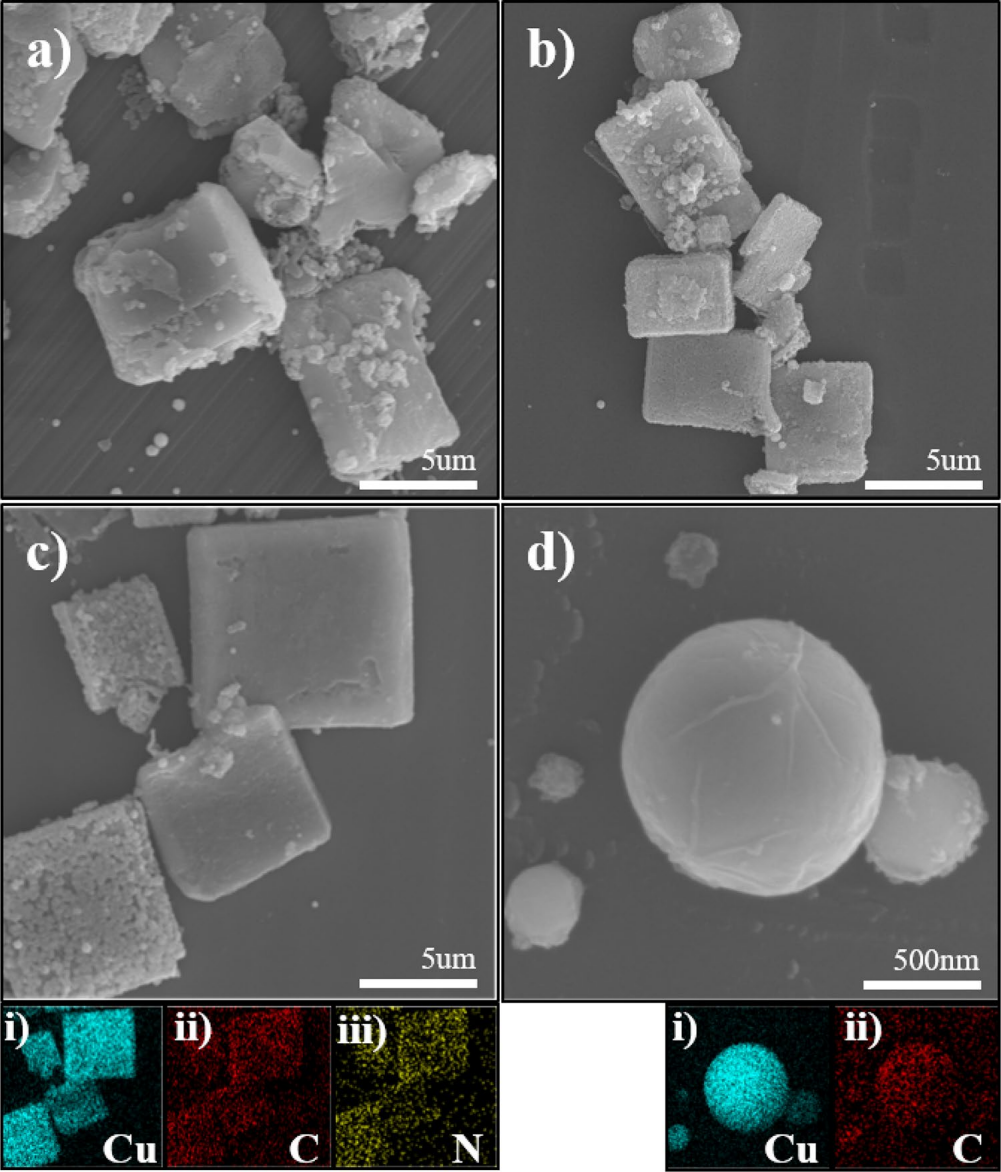
SEM images of samples prepared by PLA of a Cu plate in acetonitrile at (**a**) 355, (**b**) 532, (**c**) 1064 nm, and (**d**) spherical Cu NPs covered with carbon shells present in all of the above samples. EDS analysis of (**c**, i–iii) cubic species and (**d**, i–ii) spherical NPs.

The surface morphology and shape of the synthesized materials were studied by SEM. The SEM images of the CuCN sample synthesized in acetonitrile at different laser wavelengths are shown in Fig. 4a–c. The morphology of all samples formed in acetonitrile exhibited micro-sized cubes, which might be due to structural integrity during complex formation, and this behavior is inherent in all the metal–organic complexes^3^. The elements, from which the materials were composed, were further investigated by EDS. It was determined that the obtained microstructures consisted of Cu, C, and N (Fig. 4c(i–iii)). These results further supported the formation of CuCN in acetonitrile regardless of the laser wavelength. In addition, some spherical NPs were found among cubic CuCN (Fig. 4). The NPs were responsible for the characteristic signals of metallic Cu. Figure 4d illustrates the SEM image of spherical copper NPs obtained by PLAL of a Cu plate in acetonitrile at a wavelength of 355 nm. The EDS elemental mapping images of the sample revealed the presence of Cu and C atoms (Fig. 4d(i–ii)), which confirmed that the spherical Cu NPs were covered with carbon shells due to availability of alkyl carbons in the solvent. Thus, the graphitic carbon layers covered on the surface of micro-sized Cu spheres further prevented the aggregation and growth of the particles even after several days of aging. However, during the PLAL proceses, the micro-sized spherical particles were formed due to the Ostwald ripening process^27^. The existence of Cu@GC NPs was also confirmed by XRD and FT-Raman analyses.

**Figure 5:**
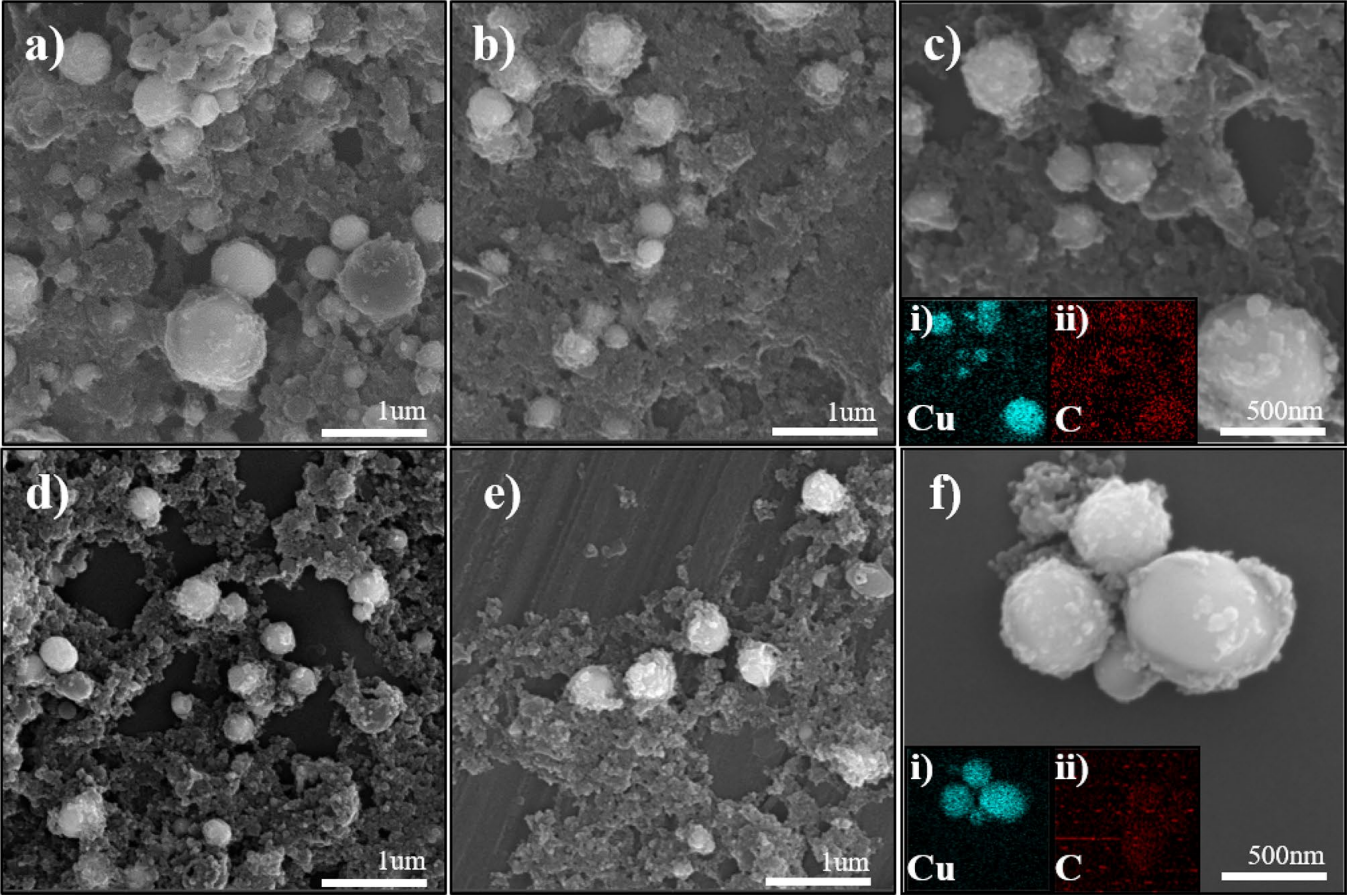
SEM images of samples synthesized in propionitrile at (**a**) 355, (**b**) 532, and (**c**) 1064 nm. (**c**, i–ii) EDS analysis of the corresponding sample. SEM images of the material prepared in butyronitrile at (**d**) 355, (**e**), 532, and (**f**) 1064 nm. (**f**, i–ii) EDS analysis of the particles prepared in butyronitrile.

Furthermore, the SEM images of samples prepared in propionitrile and butyronitrile are shown in Fig. 5. In contrast to the surface morphology of the material obtained in acetonitrile, in the case of the samples prepared in propionitrile and butyronitrile, the majority of the particles were spherical and no cubical structures were observed. It was found that the final shape of the particles in propionitrile or butyronitrile was not affected by the laser wavelength. The EDS analysis (Fig. 5c(i–ii),f(i–ii)) confirmed that similarly to the spherical particles found in acetonitrile, the spherical NPs in the samples prepared in propionitrile or butyronitrile were composed of Cu and C. Notably, the amount of Cu@GC NPs in propionitrile was significantly higher than in butyronitrile^28^.

### TEM analysis

**Figure 6:**
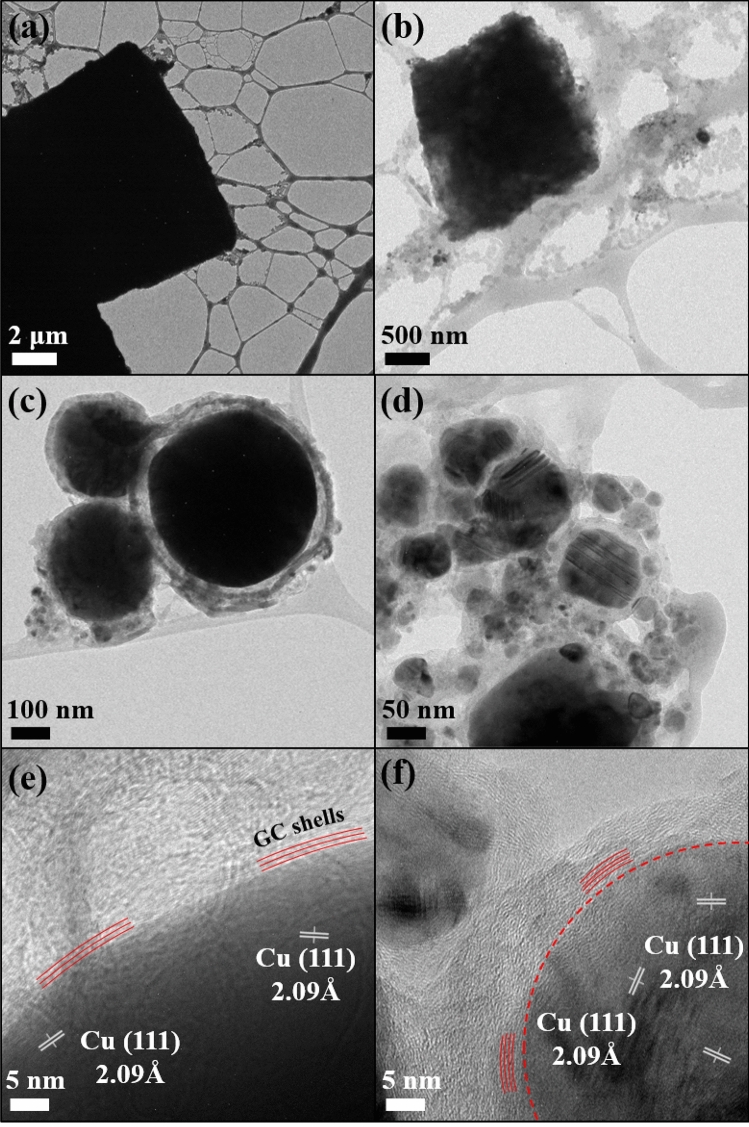
TEM images of (**a**,**b**) CuCN and (**c**-**f**) Cu@GC prepared by PLAL at 1064 nm wavelength.

TEM analysis was performed to further confirm the surface structure of the samples prepared by PLAL with 1064 nm, and the resulting images are shown in Fig. 6. The images of Cu@GC in Fig. 6c–f are clearly demonstrated the carbon layers (red lines) covered on the surface of Cu spheres with a lattice distance of 3.35 Å. The carbon shells appeared as thick as the previous works with Au@GC and Pt@C particles obtained by PLAL^8,11,29^.

### Theoretical calculations

Experimental analysis confirmed the formation of CuCN only in acetonitrile, which was supported by the conducted theoretical studies. Overall, analysis of all of the experimental results was in full agreement with the theoretical calculations.

**Figure 7:**
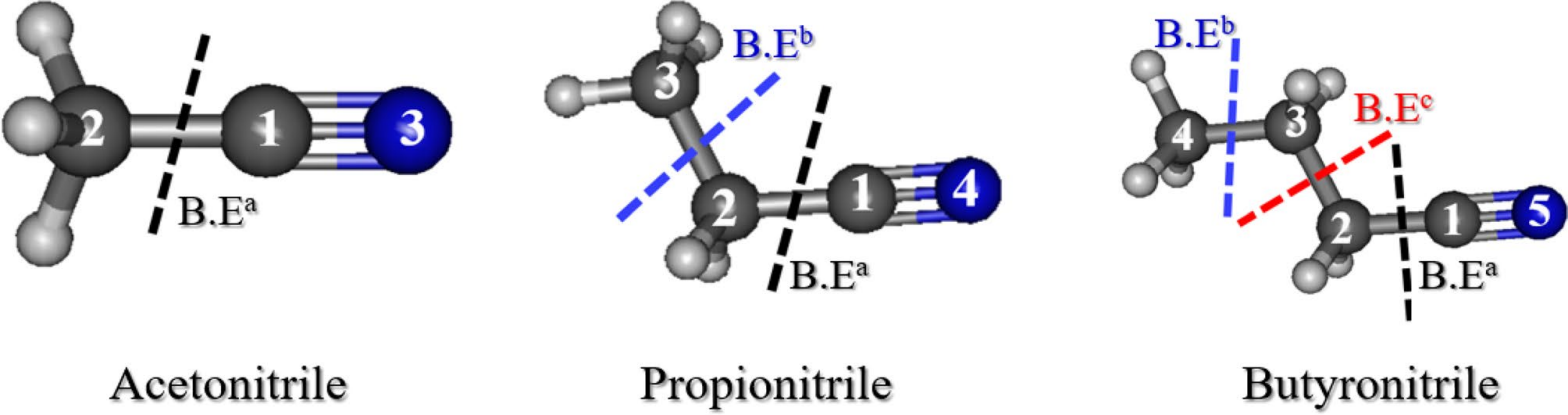
Structures of organic nitrile solvents showing the separated bonds, for which the binding energy (BE) was calculated. BE^a^—bond between the C_1_ and C_2_ atoms (C_1_–C_2_ in all nitriles), BE^b^—bond between two carbon atoms (C_2_–C_3_ in propionitrile or C_3_–C_4_ in butyronitrile), BE^c^—bond between the C_3_ and C_2_ carbon atoms (C_3_–C_2_) in butyronitrile.

During the PLALprocesses, particle formation and organic solvent decomposition occur simultaneously under the harsh conditions of the plasma plume. The difference in metal source and liquid composition can lead to a variety of materials^29^. Hence, the fragmentation of solvent molecules in the PLAL process occurs due to the difference in binding energy between atoms, considered as a solvent effect. Based on the theoretical calculations summarized in Table 1, it was clear that the formation of CuCN was favorable in acetonitrile, due to the presence of cyano groups immediately after fragmentation of the solvent molecules. As the bond between C_1_ and C_2_ dissociated, a methyl cation (CH_3_^+^) and a cyanide anion (CN^−^) were formed (Fig. 7). The electron configuration of Cu is [Ar]3d^10^4s^1^, where the filled d-subshell promotes the formation of stable complexes with ligands by π-back bonding^22^. The back donation of the ligand to the metal through sigma (σ) bonding and further transfer of the electron density back into the π orbitals of the ligand have also been reported^30^. The CN^−^ species are entirely negatively charged due to the presence of a lone pair of electrons on either side of the atom. Consequently, a strong complexation occurs between CN^−^ and Cu^+^. Moreover, most of the Cu ions produced during PLAL bind to cyano groups due to the strong affinity of these moieties for each other. This is caused by d_π_–p_π*_ back donation of the d^10^ Cu(I) orbitals to the low-lying π* orbitals of CN^−^^31^. In addition to the formation of CuCN in acetonitrile, some spherical Cu NPs covered with GC shells were observed due to the presence of methyl groups in the solvent. Nonetheless, the carbon source was insufficient to produce many spherical Cu@GC NPs in acetonitrile.

**Table 1:** Binding energies (C–C bond) calculated for the studied organic nitriles (kJ/mol).

Method	Acetonitrile	Propionitrile		Butyronitrile		
	BE^a^	BE^a^	BE^b^	BE^a^	BE^b^	BE^c^
B3LYP	530.78	516.14	342.55	518.87	381.16	329.45
MP2	611.63	609.42	421.37	612.89	415.04	421.12
CCD	607.63	602.88	378.64	605.71	389.39	375.7
CCSD	510.53	505.1	358.08	507.73	388.31	354.49
CAM-B3	544.46	534.92	356.41	538.49	391.88	345.23
M062X	552.47	542.47	379.86	545.23	410.43	372.75
LC-wPBE	553.16	581.59	365.2	544.86	394.39	355.29
LC-BLYP	576.29	566.59	380.16	571.38	411.78	371.35
M052X	564.94	554.55	376.66	557.84	410.94	368.99

*BE^a^ : C_1_–C_2_ Binding energy^a^.

*BE^b^ : C_2_–C_3_ (C_3_–C_4_) Binding energy^b^.

*BE^c^ : C_3_–C_2_ Binding energy^c^.

*Basis set: 6-311++ G(d,p).

**Figure 8:**
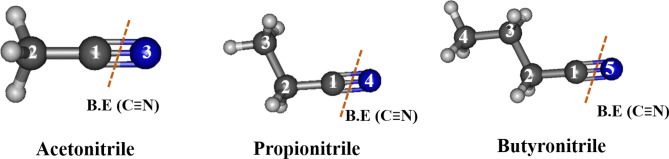
Structure of organic nitrile solvents showing the separated C≡N bonds, for which the binding energy was calculated.

Another interesting observation was made when propionitrile and butyronitrile were used as solvents for PLAL of Cu plate. Structural analysis revealed the formation of spherical NPs composed of Cu and C. The characteristic peaks of Cu as well as D- and G-bands of GC were detected in the XRD patterns and FT-Raman spectra of these samples, respectively. Moreover, the characteristic peaks corresponding to CuCN were not detected in the following spectra, and no traces attributed to cubic particles were observed during the FE-SEM analysis. To explain these outcomes, we conducted theoretical calculations of the binding energies (Table 1). Bonds between carbon atoms C_1_–C_2_ and C_2_–C_3_ were considered (Fig. 7). Both C_1_–C_2_ and C_2_–C_3_ exhibit distinct binding energy (BE^a^ and BE^b^, respectively). The values of BE^b^, i.e., the energy between the C_2_ and C_3_ atoms in propionitrile, and BE^c^ in butyronitrile were lower. These bonds underwent dissociation during PLAL, releasing a large amount of alkyl carbons, which further deposited on the surface of Cu NPs to form GC layers. Once Cu NPs were encapsulated in carbon shells, further interactions with the cyano groups were not possible. The GC layers hindered the growth of Cu NPs and prevented external impact, enhancing the stability of the material. Thus, the shapes of all particles synthesized in propionitrile and butyronitrile were spherical. Accordingly, the formation of CuCN upon the elongation of the carbon chain in organic nitriles was less likely. It was also confirmed that the binding energies of C–C and C≡N in acetonitrile were 530.78 and 1167.64 kJ/mol. Hence, it was determined that the C≡N bond was stronger than the C–C bond (Fig. 8, Table 2). Consequently, it was predicted that CuCN was formed by the cleavage of the C–C bond in acetonitrile during PLAL. The theoretical calculations of the bond energies fully supported the experimental results of this study on the predominance of CuCN in acetonitrile and the absence of CuCN in the other two nitriles through comparison.

**Table 2:** Binding energies (C≡N bond) calculated for various organic nitrile solvents (kJ/mol).

	C≡N binding energy (kJ/mol)		
	Acetonitrile	Propionitrile	Butyronitrile
B3LYP	1167.64	1166.88	1167.51
MP2	1249.62	1250.38	1250.82
CCD	1117.79	1117.78	1118.03
CAM-B3	1172.4	1175.78	1176.43
M062X	1192.31	1191.99	1192.36

*C≡N binding energy (kJ/mol).

*Basis set: 6-311++ G(d,p).

Three different laser wavelengths (1064, 532, and 355 nm) were used for the material preparation in three different organic nitriles (acetonitrile, propionitrile, and butyronitrile). The basic differences among the laser wavelengths are as follows. When a focused beam of 1064 nm wavelength is used in PLAL, the temperature of plasma plumes is higher than the case of 532 and 355 nm, while the electron densities in the plasma plume are higher at shorter wavelength. In addition, in the case of ablation at 355 nm, the plasma plume size is smaller and the expansion rate is slower^32–34^. Based on these behaviors, unique results were obtained by the formation of CuCN when using acetonitrile as a solvent at all three wavelengths due to its lower carbon content than the other solvents and direct presence of CN^−^ groups after solvent fragmentation. On the other hand, in the case of propionitrile and butyronitrile, CuCN was not formed even at high electron density of laser ablation because the binding energy of BE^b^ or/and BE^c^ is much lower than that of BE^a^ (Fig. 7 and Table 1). Thus, the formation of carbon layers is much more favorable than that of CN^−^ groups, which inhibits the formation of CuCN in propionitrile and butyronitrile.

## Conclusions

In summary, a facile one-pot synthesis of CuCN was performed using PLAL in acetonitrile. Theoretical studies on the formation of CuCN were conducted using quantum chemical calculations by performing basic sets of B3LYP and MP2. It was found that the formation CuCN was favorable only in acetonitrile, which was attributed to the high availability of the CN^−^ moiety immediately following the fragmentation of acetonitrile under PLAL. In contrast, the use of highly ordered nitrile solvents, such as propionitrile and butyronitrile, resulted in a release of a high amount of alkyl carbons, which further deposited on the surface of Cu NPs to form GC layers (Cu@GC). The formation of cubic CuCN and spherical Cu@GC structures was examined by XRD, FT-Raman, FE-SEM, EDS, and TEM. The results of the present study involving PLAL-assisted synthesis of CuCN with a unique surface structure can be utilized for the development of novel metal–polymer materials for applications in various fields.

## Data Availability

The data which support for this research findings are available and can provided based on the request to the corresponding authors.
